# Moral identity in relation to emotional well-being: a meta-analysis

**DOI:** 10.3389/fpsyg.2024.1346732

**Published:** 2024-03-12

**Authors:** Marlon Goering, Carlos N. Espinoza, Alecia Mercier, Emma K. Eason, Charles W. Johnson, Caroline G. Richter

**Affiliations:** Department of Psychology, University of Alabama at Birmingham, Birmingham, AL, United States

**Keywords:** moral identity, emotional well-being, meta-analysis, individualism, collectivism

## Abstract

**Introduction:**

This meta-analytic review surveyed previous empirical studies that examined links between moral identity and indicators of emotional well-being. Additionally, this meta-analysis examined cultural origin as a moderator, testing if links between moral identity and emotional well-being differ in collectivistic vs. individualistic countries.

**Methods:**

A systematic literature review was conducted through ProQuest’s 65 databases and PubMed. A random-effect meta-analysis and subgroup analyses were conducted using Comprehensive Meta-Analysis 4.0 (CMA) software.

**Results:**

Drawing on 27 eligible studies, moral identity was associated with greater emotional well-being (*r* = 0.27, *p* < 0.001). Follow up analyses on individual dimensions showed medium effect sizes in links between moral identity and greater happiness or positive affect (*r* = 0.28, *p* < 0.001), greater sense of purpose or meaning in life (*r* = 0.29, *p* < 0.001), and higher self-esteem (*r* = 0.25, *p* < 0.001). Moreover, moral identity was associated with greater life satisfaction showing a small effect size (*r* = 0.15, *p* = 0.011). Results showed that effect sizes of links between moral identity and overall emotional well-being did not significantly differ by cultural origin. However, effect sizes tended to be larger in the nine studies that were conducted in collectivistic countries (*r* = 0.30, *p* < 0.001) as compared to the 15 studies that were conducted in individualistic countries (*r* = 0.27, *p* < 0.001).

**Discussion:**

The results of this meta-analysis indicate a robust empirical relationship between moral identity and emotional well-being that is present across various dimensions of emotional well-being and in both individualistic and collectivistic cultures.

**Systematic review registration:**

https://osf.io/94f8b/?view_only=6db54da0fa304c83993d0438ecb5c637

## Introduction

The idea that a person’s moral self and emotional well-being are connected is ancient. In the opening book of the Nicomachean Ethics, the Greek philosopher Aristotle postulates that living a morally virtuous life is equivalent to living a happy life (Aristotle, ca. 350 BCE/ Bartlett and Collins, 2011), meaning that pursuing the moral good and focusing on developing a good character would lead to emotional well-being in form of eudaimonia (McBeath and Webb, 2002). The same idea was present in ancient eastern cultures with Confucianism emphasizing that an individual’s moral virtues and emotional well-being are connected (Kim, 2020). The degree to which being a “moral person” is important to an individual can be measured as their level of moral identity (Hardy and Carlo, 2011), which describes how central moral values and concerns are to a person’s sense of self (Aquino and Reed, 2002). In other words, individuals with high moral identity consider moral values such as being fair, caring, compassionate, and honest as important aspects of their self-concept.

The concept of moral identity was initially introduced to bridge the moral judgment-action gap by mediating links between moral reasoning and moral behavior (Blasi, 1983). Specifically, it has been proposed that individuals who consider moral values as central to their self-identity would be less likely to commit moral transgressions as this would imply a violation of one’s self concept. Indeed, previous research has linked moral identity with greater moral integrity (Krettenauer et al., 2022), which in turn has been linked with greater emotional well-being (Olson, 2002). Relatedly, empirical research has shown a robust relationship between moral identity and moral behavior (Hertz and Krettenauer, 2016) as well as prosocial and altruistic behaviors (Patrick et al., 2018). Engaging in moral and prosocial behaviors does not only benefit others but also leads to positive emotions in those performing the behavior (Miles and Upenieks, 2022). For example, a meta-analysis has found that volunteering is associated with greater emotional well-being (Jenkinson et al., 2013). Associations between prosocial behavior and greater emotional well-being may be due to positive environmental responses (Jongenelis and Pettigrew, 2021) as well as internal emotional rewards such as feeling a “warm glow” following the behavior (Andreoni, 1989) and being pleased with one’s character (McKinnon, 2005).

Other mechanisms through which moral identity may relate to emotional well-being include greater self-consistency and autonomy. A few studies have reported that individuals with high levels of moral identity show greater self-consistency in values and behaviors (Van Gils and Horton, 2019). According to early personality theories, self-consistency is an elemental human motive (Lecky, 1945) and predicts emotional well-being by reducing internal tensions and providing stable direction and coherence to one’s life (McKinnon, 2005). Indeed, empirical research has supported links between self-consistency and greater emotional well-being (Cross et al., 2003). Ultimately, high moral identity is indicative of autonomy in making identity commitments and associated with achieving a mature sense of self (Hardy et al., 2013). A sense of agency in identity formation and reaching identity achievement have in turn been linked with greater emotional well-being (Burrow et al., 2010; Sandhu et al., 2012). Thus, moral identity may contribute to greater emotional well-being through higher engagement in moral and prosocial behavior, greater integrity and self-consistency, as well as autonomy in forming an identity.

In addition to the aforementioned framework on how moral identity may contribute to emotional well-being, emotional well-being may also contribute to higher moral identity. It is possible that unsatisfied emotional needs can leave individuals preoccupied with personal stress, which in turn may delimit capacity to concern for moral issues and needs of others (Nitschke and Bartz, 2022). Higher emotional well-being may therefore allow greater capacity for developing a moral identity. Thus, the association between moral identity and greater emotional well-being may be bidirectional and any directional inferences from cross-sectional research must be made with caution.

Contrary to the framework proposing that moral identity is linked with greater emotional well-being, previous studies have shown that moral identity can increase negative self-evaluative moral emotions (Lefebvre and Krettenauer, 2019). Specifically, individuals with high moral identity are more likely to experience guilt (Ding et al., 2016) and shame (Abraham and Berline, 2015), which in turn diminish emotional well-being (Velotti et al., 2017). When external circumstances compel individuals with high moral identity to compromise or violate their moral principles it can pose a major threat to their emotional well-being (McKinnon, 2005). Moreover, moral identity makes individuals more sensitive and emotionally susceptible to systemic and situational injustices (O’Reilly et al., 2016), which may in turn reduce emotional well-being.

Existing meta-analytic reviews have investigated links between moral identity and moral behavior (Hertz and Krettenauer, 2016) as well as moral identity and moral emotions (Lefebvre and Krettenauer, 2019). Likewise, previous reviews have examined links between emotional well-being and prosociality (Hui et al., 2020) and investigated emotional well-being in relation to aspects of personality and identity such as the Big-Five personality types (Anglim et al., 2020) and racial/ethnic identity (Smith and Silva, 2011). However, no previous study has synthesized the literature on links between moral identity and non-moral emotional well-being. Thus, a systematic review and meta-analysis examining the direction and magnitude of links between moral identity and emotional well-being is needed.

### Emotional well-being

The empirical literature includes varying terminologies aimed at operationalizing emotional well-being. For example, some working terminologies for emotional well-being have included terms such as flourishing, subjective well-being, and psychological well-being each of which may or may not include mental health as part of its definition (Diener et al., 1985; Ryff and Keyes, 1995; Seligman, 2012). However, there have been efforts to unite the field under the term emotional well-being, which specifically excludes mental health conditions such as depression and anxiety as indicators of emotional well-being (Feller et al., 2018). These efforts have resulted in a working definition of emotional well-being as “a multi-dimensional composite that encompasses how positive an individual feels generally and about life overall. [….] These features occur in the context of culture, life circumstances, resources, and life course.” (Park et al., 2023). Variables that encompass various dimensions of emotional well-being include happiness, life satisfaction, meaning in life, and self-esteem. For example, happiness is an emotionally positive state of being (Elfenbein and Ambady, 2002), life satisfaction and meaning in life are general appraisal of one’s existence (Pavot and Diener, 1993; Steger et al., 2006), and self-esteem is a positive evaluation of self-worth (Abdel-Khalek, 2016). These dimensions have all been previously included together in meta-analyses focusing on emotional well-being (e.g., Schmitt et al., 2014; Çikrıkci, 2016; Tang et al., 2020).

### Moral identity and dimensions of emotional well-being

There are good conceptual reasons for why moral identity is related to each of these four dimensions of emotional well-being. One way how moral identity may relate to happiness, life satisfaction, meaning in life, and self-esteem is through engagement in prosocial behavior. Previous research has shown robust links between moral identity and prosocial behavior (Hertz and Krettenauer, 2016), which in turn has been linked with greater happiness (Aknin and Whillans, 2021), life satisfaction (Espinosa et al., 2022), meaning in life (Klein, 2017), and higher self-esteem (Fu et al., 2017). While these four dimensions of emotional well-being are intercorrelated (Lin et al., 2021), they differ in their cognitive, emotional, and reflective processes. Self-esteem and meaning in life both involve reflective self-evaluations with self-esteem involving internal beliefs toward oneself (Schmitt et al., 2014) and meaning in life involving cognitive reflections about the value one attributes to one’s existence (Dezutter et al., 2013). In comparison, happiness and life satisfaction are mainly based on lived experiences, with happiness being based on positive hedonic emotions and life satisfaction involving a cognitive assessment of the extent to which life experiences satisfy an individual’s desires (Schmitt et al., 2014).

These differences may affect the specific ways how moral identity relates to each dimension of emotional well-being. For example, moral identity may be related to higher self-esteem primarily through a more positive moral self-evaluation, which may be gained from a conscious sense of moral integrity that is present in individuals with high moral identity (Krettenauer et al., 2022). Conversely, moral identity may relate to greater purpose and meaning in life by providing a direction and ideals to strive for (Higgins-D’Alessandro and Power, 2005). In contrast, happiness and life satisfaction may require less self-evaluation and can be gained directly from positive experiences. While happiness and positive affect may be purely emotional responses, life satisfaction involves a conscious and reflective judgment of dispositional emotional well-being (Huebner and Dew, 1996). Having a conscious sense of moral integrity may therefore be more relevant for links between moral identity and life satisfaction, whereas moral identity may relate to happiness primarily through the emotional benefits of more frequently engaging in moral and prosocial behavior (Miles and Upenieks, 2022). Given these potential differences in the underlying mechanisms, it is possible that links between moral identity and these sub-dimensions differ in magnitude, which underlines the importance of examining heterogeneity in effects sizes of moral identity and emotional well-being across these dimensions. Thus, this study sought to explore moral identity and its relationship with overall emotional well-being as well as its individual relationship with happiness or positive affect, life satisfaction, meaning or purpose in life, and self-esteem.

### Cultural origin

Previous meta-analyses focusing on moral identity noted the importance of examining cultural differences, especially differences between individualism and collectivism (Hertz and Krettenauer, 2016; Lefebvre and Krettenauer, 2019). While individualistic cultures stress independence and autonomous self-actualization, collectivistic cultures are characterized by interdependence and individuals putting greater emphasis on needs of their community when constructing a self-identity (Markus and Kitayama, 1991). Although the general concept of moral identity is similar across cultures, the degree to which specific moral values are accentuated varies between individualistic and collectivistic cultures (Jia et al., 2019). The existing and most-widely used measures of moral identity may neglect moral attributes that are specific to eastern-collectivistic cultures, which possibly introduces cultural-bias into the concept of moral identity (Jia and Krettenauer, 2017). This potential bias has been referred to as an explanation for weaker links between moral identity and moral behavior in collectivistic cultures as compared to individualistic cultures (Hertz and Krettenauer, 2016). However, a previous meta-analysis examining moral identity in relation to moral emotions showed no differences between individualistic and collectivistic cultures (Lefebvre and Krettenauer, 2019).

No previous studies have examined cultural differences in links between moral identity and emotional well-being. There is some theoretical framework suggesting that links between moral identity and emotional well-being may be stronger in collectivistic cultures as compared to individualistic cultures. Due to greater cultural emphasis on independence and individual achievement (Markus and Kitayama, 1991), individuals from individualistic cultures experience stronger positive emotions in response to personal success, whereas individuals from collectivistic cultures have stronger positive emotions in response to outcomes that benefit others (Stipek, 1998). Since moral identity implies integrating personal motives with other-oriented moral principles (Frimer and Walker, 2009), moral identity may play a more crucial role for emotional well-being in collectivistic as compared to individualistic cultures. Thus, this study sought to examine whether links between moral identity and overall emotional well-being differ between individualistic and collectivistic cultures.

## The current review

The central aim of this study was to systematically review and meta-analyze existing empirical studies on the relationship between moral identity and emotional well-being. Since emotional well-being includes several dimensions, the present study also conducted meta-analyses of links between moral identity and each of four sub-dimensions of emotional well-being (i.e., happiness/positive affect, sense of purpose/meaning in life, life satisfaction, and self-esteem). A second aim of the present study was to investigate whether links between moral identity and emotional well-being differ in magnitude between collectivistic and individualistic cultures. It was hypothesized that higher moral identity is associated with higher emotional well-being. Moreover, it was hypothesized that links between moral identity and emotional well-being are stronger in studies conducted in collectivistic countries as compared to studies conducted in individualistic countries.

## Methods

**FIGURE 1:**
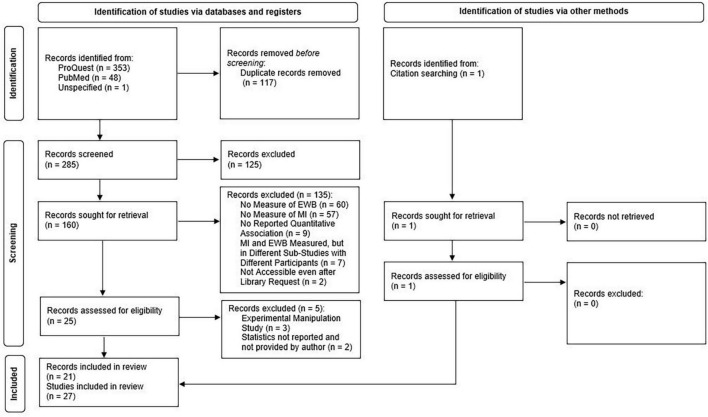
PRISMA 2020 flow diagram for the systematic search on moral identity and emotional well-being (Page et al., 2021a).

This meta-analysis was pre-registered in the Open Science Framework (OSF). A literature search was conducted following the PRISMA 2020 guidelines for systematic reviews (Page et al., 2021a), see Figure 1 for the complete flow diagram. The pre-registration and data are available at OSF.^1^

### Search strategy

The literature searches for studies that examined links between moral identity and emotional well-being were conducted through ProQuest with its 65 databases including APA PsycInfo, APA PsycArticles, ERIC, ProQuest Dissertations & Theses Global, and Sociological Abstracts, among others. A separate literature search was conducted through PubMed. The literature search included two groups of key-terms. Group A included moral identity and Group B included various terms and phrases indicating emotional well-being (see Table 1 for details). Articles that included moral identity and at least one of the emotional well-being terms were captured. Moral identity was included as a single search term, following methods of previous meta-analyses that examined links between moral identity and moral behavior or moral emotions (Hertz and Krettenauer, 2016; Lefebvre and Krettenauer, 2019). Similarly, emotional well-being terms were based on the working definition by Park et al. (2023) and adapted from Koslouski et al. (2022).

**TABLE 1:** Search terms used for ProQuest.

Group A	all(moral NEAR identit*)
Group B	[all(emotional NEAR well-being) OR all(emotional NEAR wellbeing) OR all(emotional NEAR well NEAR being) OR all(eudemonic NEAR well-being) OR all(eudemonic NEAR wellbeing) OR all(eudemonic NEAR well NEAR being) OR all(eudaimoni*) OR all(quality NEAR life) OR all(QOL) OR all(EWB) OR all(psychological NEAR well-being) OR all(psychological NEAR wellbeing) OR all(psychological NEAR well NEAR being) OR all(life NEAR satisfaction) OR all(satisfaction) OR all(satisfied) OR all(happiness) OR all(subjective NEAR well-being) OR all(subjective NEAR wellbeing) OR all(subjective NEAR well NEAR being) OR all(happy) OR all(positive NEAR emotion) OR all(evaluative NEAR well-being) OR all(evaluative NEAR wellbeing) OR all(evaluative NEAR well NEAR being) OR all(hedonic NEAR well-being) OR all(hedonic NEAR wellbeing) OR all(hedonic NEAR well NEAR being) OR all(experiential NEAR well-being) OR all(experiential NEAR wellbeing) OR all(experiential NEAR well NEAR being) OR all(meaning NEAR life) OR all(positive NEAR affect) OR all(self-esteem) OR all(selfesteem) OR all(self NEAR esteem) OR all(self-worth) OR all(selfworth) OR all(self NEAR worth)]

Group A and B were be combined with AND. The statement “NEAR” includes results where terms from each group appear within four words of proximity in the manuscript.

*Allows for flexible word endings. The same search terms were used for the PubMed search but the code was adapted to meet PubMed search rules.

All searches were limited to peer-reviewed journal articles, book chapters, dissertations/theses, and conference papers. No restrictions were placed on the location of where a search term needed to appear in the manuscript. Articles that were not available in either English or Spanish were excluded. Manuscripts that were unavailable online were requested from the authors’ university library.

### Eligibility criteria

To be included in the meta-analysis, eligible studies needed to include an independent empirical assessment of moral identity defined as the degree of how central moral values and beliefs are to one’s self-identity. Eligible studies also needed to include an independent empirical assessment of emotional well-being. Studies only measuring domain-specific forms of emotional well-being (e.g., career satisfaction) were excluded. Studies were excluded if moral identity and/or emotional well-being were measured following an experimental manipulation. Other inclusion criteria were that studies needed to include quantitative results on the relationship of mortal identity and at least one dimension of emotional well-being. If results were not available in the original paper, the corresponding authors were contacted to either provide the result or statistics that allow computing it. Finally, eligible studies had to use original data. There were no restrictions on year of publication or study populations.

### Literature review

The studies identified by the literature search were reviewed following the guidelines of the PRISMA 2020 flow diagram template for systematic reviews (Page et al., 2021a). First, the number of records found were reported for each database and duplicates were removed (*n* = 117). The literature review on the articles identified in the literatures search (*n* = 286) then consisted of three stages including title and abstract screening, full-text screening, and data extraction. The procedures were conducted by the first, second, fourth, and fifth author at each stage through Covidence (systematic review software; Veritas Health Innovation, 2023). Each record was reviewed independently by at least two reviewers at each stage of the review process to ensure blinding. At each stage, conflicts between reviewer assessments were resolved by the first and second author through discussion until consensus was reached. At the first stage, all records were screened by reviewing the title and abstract. Records were excluded when not reporting quantitative results or when not investigating any of the variables of interest meaning that records were only excluded in the first round if the title and/abstract neither mentioned that moral identity nor a dimension of emotional well-being were measured in the study. Inter-rater agreement between the reviewers at the title and abstract screening stage was fair (72% agreement, Cohen’s *k* = 0.38). As illustrated in Figure 1, the title and abstract screening resulted in 161 records proceeding to the full-text screening stage. At the second stage, full-text reviews were conducted and records were excluded if they did not include a quantitative measure of both moral identity and an indicator of emotional well-being in the same study. Inter-rater agreement between the reviewers at the full-text screening stage was almost perfect (94% agreement, Cohen’s *k* = 0.81). The full-text reviews resulted in 25 records proceeding to the third stage. At the third stage, the remaining records were assessed for eligibility and three records were excluded due to involving experimental manipulations prior to the assessments of moral identity and emotional well-being, which may have affected their association (Aquino et al., 2011; Furukawa et al., 2021; Semko, 2022). Moreover, two records were excluded for which results on links between moral identity and emotional well-being could not be obtained from the manuscript or received from the corresponding authors. Finally, the reference lists of the articles that were included after the full-text screening were examined. This citation searching resulted in one additional study being included in the review resulting in a total of 21 records being included in the review. Of these 21 records, three records included two eligible studies with separate samples (Giacalone et al., 2016; Wu et al., 2020; Wang and Hackett, 2022) and one record included four eligible studies with four separate samples (Jordan et al., 2015). Thus, the literature review yielded 27 eligible studies that were included in the meta-analysis.

### Data extraction

From the eligible studies, the first and second author independently extracted key study characteristics. These include information on sample size and sample characteristics, information on the measures that were used for moral identity and emotional well-being, and effect sizes of relationships between moral identity and emotional well-being variables. The authors extracted correlation coefficients (*r*) from each individual study as the effect size. Three studies reported latent correlations between moral identity and emotional well-being, which were used for the analyses. When necessary, effect sizes were reverse coded so that positive correlation coefficients indicate that higher moral identity is associated with greater emotional well-being. Discrepancies between the two authors were resolved through discussion until consensus was reached.

The most commonly used measure of moral identity among the eligible studies was the Self-Importance of Moral Identity Scale (SIMI) (Aquino and Reed, 2002). This scale includes two subdimensions – internalization and symbolization. While the internalization dimension measures how central moral traits are to one’s self-concept, the symbolization dimension measures the degree to which individuals engage in public behavior to demonstrate their moral traits (Aquino and Reed, 2002). Since the focus of the present study is on moral identity defined as how central moral traits are to one’s self-identity, the internalization scale was prioritized. Therefore, if studies reported effects sizes for both the internalization and symbolization subscales separately, only the effect sizes for internalization were used in the analyses. However, if studies only reported effect sizes of the overall moral identity scale (i.e., combining internalization and symbolization), these effect sizes were used for the analyses. This approach is consistent with previous meta-analyses involving moral identity (Hertz and Krettenauer, 2016; Lefebvre and Krettenauer, 2019). Measures of emotional well-being were categorized into four groups (i.e., life satisfaction, sense of purpose/meaning, happiness/positive affect, and self-esteem). For studies that included multiple measures of emotional well-being, effect sizes of moral identity with the individual emotional well-being measures were combined to a single effect size if the measures of emotional well-being were in the same category. For example, effect sizes of the correlations of moral identity and happiness and of moral identity and positive affect in the same study were averaged.

### Coding of cultural origin as a moderator

Each eligible study was coded as either having involved sampled participants from individualistic or collectivistic cultures based on the country of data collection using data from a revised version of Hofstede’s quantitative measure of individualism (Minkov and Kaasa, 2022). This quantitative measure of individualism was initially developed from survey responses of employees of the same company from various countries to create country-level indicators of individualism (Hofstede, 1980). In countries that have a higher Individualism index score (IDV), people on average put greater emphasis on achieving personal goals, while in countries with lower IDV scores, people on average put greater emphasis on goals of larger groups and communities (Hofstede and McCrae, 2004). While the Hofstede measure has been criticized for being limited to the work environment, previous research has demonstrated convergent validity of the IDV and other measures of individualism (Schimmack et al., 2005).

Each study was assigned the country-specific scores as of 2023 that were retrieved from the website of Hofstede insights and are based on more recent replications of Hofstede’s initial study (Country Comparison Tool, 2023).^2^ The majority of studies included in this meta-analysis were conducted in the United States (12 studies, IDV = 91) and China (6 studies, IDV = 20). Following Hofstede’s classification, the United States studies as well as three studies involving participants from both the United States and Canada (IDV = 81) were coded as individualistic. Studies conducted in China as well as a study conducted in Indonesia (IDV = 14), a study conducted in Iran (IDV = 41), and a study conducted in Georgia (IDV = 41) were coded as collectivistic. Two studies involved international business students and one study recruited participants via Amazon Mechanical Turk with no indication of geographical restrictions. These studies were not coded for cultural origin of the sample.

### Data analysis

Data were extracted from the final included articles and uploaded into Comprehensive Meta-Analysis 4.0 (CMA) software (Borenstein et al., 2022) to conduct the meta-analysis. Before the calculation of the pooled effect size, we transformed correlation coefficients into Fisher’s *z* to stabilize variance in the analyses and then converted back into correlation coefficients (*r*) for ease of interpretation. Correlation coefficients can be interpreted using the following criteria: small (0.10), medium (0.24), and large (0.37) (Cohen, 1988). To address the first aim, a meta-analysis was conducted in two steps. First, an overall effect size was computed to analyze the association between moral identity and emotional well-being. Next, four separate meta-analyses were conducted across emotional well-being domains to examine the relation of moral identity with each of the four subdomains of emotional well-being. Positive effect sizes indicate that higher moral identity is related to better emotional well-being, whereas negative effect sizes indicate that higher moral identity is related to lower emotional well-being. Because it is implausible to assume that the true effect size is homogenous across the included studies, a random-effects model was used to conduct all analyses and interpret the effect sizes (Borenstein et al., 2021).

#### Subgroup analysis

To address aim two, a subgroup analysis was conducted in CMA to examine if the relationship between emotional well-being and moral identity differs between individualistic and collectivistic cultures. Differences were examined using a *Q*-test with a significant *Q*-test suggesting that the overall effect sizes differed between sampled participants from individualistic and collectivistic countries.

#### Heterogeneity

Heterogeneity between effect sizes was examined using *I*^2^, *Q*, and tau-squared (τ^2^) statistics. The *I*^2^ value quantified the percentage of variation in effect sizes due to heterogeneity and is typically categorized as low (25%), moderate (50%), or high (75%) (Higgins et al., 2003), whilst τ^2^ measured between-study variance and is the estimate of the distribution of true effects across the population of studies (Borenstein et al., 2021). The *Q* statistics is a standardized measure used to test if heterogeneity is significant across included studies (Borenstein et al., 2021).

#### Sensitivity analysis

Sensitivity analyses were conducted to investigate the robustness of the results if alternative analytic decisions were made. The first set of sensitivity analyses examined if results are robust when latent correlations are excluded from the analyses. Since measurement error is removed when estimating latent variables (Russell et al., 1998), it is possible that effect sizes of correlations between latent variables are larger than correlations between measured variables. Thus, in order to examine differences in effect sizes and heterogeneity results depending on whether latent correlations are included, all analyses were repeated excluding the three studies that report latent correlations between moral identity and emotional well-being (Hardy et al., 2013, 2014; Yukhymenko-Lescroart and Sharma, 2020). A second sensitivity analysis was conducted when testing differences between collectivistic and individualistic cultures. In this sensitivity analysis, effect sizes from the studies conducted in Georgia and Iran were excluded as these countries had a higher Individualism Distance Index (IDV = 41) and differ geographically from the other studies in the collectivistic group from East and Southeast Asia (IDV = 14–20). A final *post hoc* sensitivity analysis examined if the relationship of moral identity and overall emotional well-being would differ when moral identity is measured with the symbolization dimension of the SIMI instead of the internalization dimension. Specifically, effect sizes between moral identity and overall emotional well-being were estimated separately for the internalization and symbolization dimension of the SIMI among the eligible studies.

#### Risk of bias assessment

At the meta-analysis level, bias was reduced by including all possible studies through a systematic, comprehensive literature search using PRISMA guidelines (Page et al., 2021a). These included unpublished literature such as theses and dissertations as well as conference papers. Additionally, publication bias was assessed both visually and statistically. A funnel plot was first examined to visualize the relationship between study size and effect size and examine if effect sizes included in this meta-analysis are distributed symmetrically around the mean effect size. Small studies are more likely to be published when reporting larger effect sizes that reach statistical significance (Borenstein et al., 2021). Thus, publication bias may be present if the funnel plot would show that studies with large standard errors tend to have larger effect sizes. Because interpretation of the funnel plot may be subjective, Egger’s Regression test as well as Begg and Mazumdar’s rank correlation test were conducted to quantify asymmetry within the funnel plot (Begg and Mazumdar, 1994; Egger et al., 1997). Finally, classic fail-safe *N* was obtained to determine the number of studies with an effect size of zero that would be required for the present results to become non-significant at an alpha level of 0.05 (Rosenthal, 1979).

## Results

### Study characteristics

The literature search yielded a total of 27 eligible studies with a total of 24,733 participants that report at least one effect size of links between moral identity and emotional well-being (see Figure 1 for PRISMA flow diagram). Detailed information on the sample characteristics and measures of each eligible study are reported in Table 2. Most studies involved adolescents (*n* = 6), emerging adults (*n* = 11), and mid-aged adults (*n* = 17). Only a few studies included older adults (*n* = 3) and no studies specifically focused on older adult populations. As a result, one initially proposed and pre-registered hypothesis that links between moral identity and emotional well-being would be stronger in older adult populations could not be examined in the present study. Most studies included a similar number of males and females. Most eligible studies were conducted in either the United States (*n* = 12 studies) or China (*n* = 6). All but one study assessed moral identity using the SIMI (Aquino and Reed, 2002). Of these studies, most assessed internalization separately (*n* = 18) but few studies combined the internalization and symbolization subscales (*n* = 7). One study used a modified version of the SIMI (Yukhymenko-Lescroart and Sharma, 2020) and one study assessed moral identity with the Civic and Moral Identity Questionnaire (Han et al., 2019). All studies that assessed self-esteem utilized the Rosenberg’s Self-Esteem Scale (Rosenberg, 1965). All of the included studies that measured life satisfaction used either the Satisfaction with Life Scale (Diener et al., 1985) or its modified version the Temporal Satisfaction with Life Scale (Pavot et al., 1998). Studies that measured purpose and meaning in life utilized more diverse measures with the most frequently used measure being the Meaning in Life Questionnaire (Steger et al., 2006). Similarly, the included studies that measured positive affect and happiness used a variety of measures with the most frequently used being the Positive and Negative Affect Scale (Watson et al., 1988).

**TABLE 2:** Characteristics and effect sizes of studies included in the meta-analysis.

References	Sample (N)	Country (IND-COL)	Age range (mean)	Sex (% female)	MI measure	EWB measure	*EWB dimensions	Effect size
Boudlaie et al., 2022	813	Iran (41)	67% 30–45	19	SIMI – I&S (Aquino and Reed, 2002)	RSES (Rosenberg, 1965)	Self-esteem	*r* = 0.30
Cohen et al., 2014	1,514	United States (91)	18–71 (39.32)	50	SIMI – I (Aquino and Reed, 2002)	SWLS (Diener et al., 1985)	Life satisfaction	*r* = –0.01
						PANAS – P&N (Watson et al., 1988)	Positive affect	*r* = 0.20
							Pooled effect	*r* = 0.11
Cohen et al., 2011	450	United States (91)	17–50 (19.12)	53	SIMI – I (Aquino and Reed, 2002)	RSES (Rosenberg, 1965)	Self-esteem	*r* = 0.22
Cui et al., 2021	419	China (20)	16–25 (19.4)	78	SIMI – I (Aquino and Reed, 2002)	SWLS (Diener et al., 1985)	Life satisfaction	*r* = 0.13
						SPANE (Diener et al., 2009)	Positive affect	*r* = 0.20
							Pooled effect	*r* = 0.17
Fan et al., 2021	74	China (20)	18 and older	53	SIMI – I (Aquino and Reed, 2002)	PANAS – P&N (Watson et al., 1988)	Positive affect	*r* = 0.44
Garcia and Adrianson, 2023	336	Indonesia (14)	16–70 (27)	59	SIMI – I&S (Aquino and Reed, 2002)	TSWLS (Pavot et al., 1998)	Life satisfaction	*r* = 0.06
						PANAS – P&N (Watson et al., 1988)	Positive affect	*r* = 0.23
							Pooled effect	*r* = 0.17
Garcia et al., 2018	323	Not defined	(36.94)	51	SIMI – I&S (Aquino and Reed, 2002)	PERMA profiler (Butler and Kern, 2016)	Sense of meaning	*r* = 0.41
							Positive affect	*r* = 0.34
							Pooled effect	*r* = 0.38
Giacalone et al., 2016 – study 1	206	United States (91)	25% 18–25; 31% 26–35	47	SIMI – I (Aquino and Reed, 2002)	MLQ – presence (Steger et al., 2006)	Sense of meaning	*r* = 0.45
						SHS (Lyubomirsky and Lepper, 1999)	Happiness	*r* = 0.29
							Pooled effect	*r* = 0.37
Giacalone et al., 2016 – study 2	254	United States (91)	50% 18–25; 39% 26–35	56	SIMI – I (Aquino and Reed, 2002)	SWLS (Diener et al., 1985)	Life satisfaction	*r* = 0.17
Han et al., 2019	1,555	United States (91)	(17.33)	52	CIS – MI (Beaumont et al., 2006)	MLQ – presence (Steger et al., 2006)	Sense of meaning	*r* = 0.21
Han, 2022	1,079	United States (91)	(22.04)	86	SIMI – I (Aquino and Reed, 2002)	CPS – meaning (Bronk et al., 2018)	Sense of meaning	*r* = 0.11
Hardy et al., 2013	9,500	United States (91)	18–25 (19.78)	73	SIMI – I (Aquino and Reed, 2002)	RSES (Rosenberg, 1989)	Self-esteem	*r* = 0.47
						MLQ – presence (Steger et al., 2006)	Sense of meaning	*r* = 0.35
							Pooled effect	*r* = 0.41
Hardy et al., 2014	366	United States (91)	15–18 (16.3)	43	SIMI – I (Aquino and Reed, 2002)	Revised Youth Purpose Scale (Bundick et al., 2006)	Sense of purpose	*r* = 0.40
Hart et al., 2019	405	United States (91)	(38.07)	62	SIMI – I (Aquino and Reed, 2002)	RSES (Rosenberg, 1989)	Self-esteem	*r* = 0.22
Hofmann et al., 2018	758	United States (91)	(32.2)	51	SIMI – I (Aquino and Reed, 2002)	Sense of purpose single item	Sense of purpose	*r* = 0.16
						Momentary happiness single item	Happiness	*r* = 0.05
							Pooled effect	*r* = 0.11
Hu, 2022	431	China (20)	18–25	53	SIMI – I (Aquino and Reed, 2002)	Modified PWB (Ryff, 1989)	Psychological well-being	*r* = 0.38
Jordan et al., 2015 – sample 1a	544	United States (91)	(32.89)	48	SIMI – I (Aquino and Reed, 2002)	RSES (Rosenberg, 1989)	Self-esteem	*r* = 0.26
Jordan et al., 2015 – sample 1b	499	United States (91)	(35.94)	49	SIMI – I (Aquino and Reed, 2002)	RSES (Rosenberg, 1989)	Self-esteem	*r* = 0.22
						PANAS – P&N (Watson et al., 1988)	Positive affect	*r* = 0.24
							Pooled effect	*r* = 0.23
Jordan et al., 2015 – sample 4	115	Not defined	(21.06)	52	SIMI – I (Aquino and Reed, 2002)	RSES (Rosenberg, 1989)	Self-esteem	*r* = 0.00
Jordan et al., 2015 – sample 5	107	Not defined	(21.68)	48	SIMI – I (Aquino and Reed, 2002)	RSES (Rosenberg, 1989)	Self-esteem	*r* = –0.04
Mestvirishvili et al., 2020	318	Georgia (41)	16–56 (21.75)	65	SIMI – I (Aquino and Reed, 2002)	TEIQue – EWB (Martskvishvili et al., 2013)	Emotional well-being	*r* = 0.29
Wang and Hackett, 2022 – leaders sample	131	United States/Canada (86)	91% 31 or older	61	SIMI – I&S (Aquino and Reed, 2002)	PSQ (Brebner et al., 1995)	Happiness	*r* = 0.48
Wang and Hackett, 2022 – followers sample	131	United States/Canada (86)	89% 31 or older	47	SIMI – I&S (Aquino and Reed, 2002)	PSQ (Brebner et al., 1995)	Happiness	*r* = 0.44
Wu et al., 2020 – husband sample	587	China (20)	(41.37)	0	SIMI – I&S (Aquino and Reed, 2002)	SWLS (Diener et al., 1985)	Life satisfaction	*r* = 0.28
Wu et al., 2020 – wife sample	587	China (20)	(40.58)	100	SIMI – I&S (Aquino and Reed, 2002)	SWLS (Diener et al., 1985)	Life satisfaction	*r* = 0.28
Yukhymenko-Lescroart and Sharma, 2020	403	United States (91)	22–81 (32.75)	66	SIMI – adapted (Aquino and Reed, 2002)	SOPS-2 (Sharma and Yukhymenko-Lescroart, 2019)	Sense of purpose	*r* = 0.27
Zuo et al., 2016	2,828	China (20)	13–19 (15.61)	56	SIMI – I (Aquino and Reed, 2002)	RSES (Rosenberg, 1989)	Self-esteem	*r* = 0.39

MI, moral identity; EWB, emotional well-being; SIMI, The Self Importance of Moral Identity Scale (I, internalized dimension; S, symbolization dimension); CIS-MI, Moral Identity Subscale of the Civic Identity Scale; RSES, The Rosenberg Self-Esteem Scale; PANAS, The Positive and Negative Affect Schedule (P, positive affect; N, negative affect); SWLS, The Satisfaction with Life Scale; SPANE, The Scale of Positive and Negative Experience; TSWLS, The Temporal Satisfaction With Life Scale; PERMA, Positive emotion, Engagement, Relationships, Meaning, Accomplishment; MLQ, Meaning in Life Questionnaire; SHS, The Subjective Happiness Scale; CPS, The Claremont Purpose Scale; PWB, Psychological Well-Being Scale; TEIQue, The Trait Emotional Intelligence Questionnaire; PSQ, The Personal State Questionnaire; SOPS-2, The Revised Sense of Purpose Scale. *Multiple effect sizes of the same emotional well-being dimension were combined (i.e., positive and negative affect).

Each study reported correlation coefficients as the effect size to describe the relationship of moral identity and emotional well-being with three studies reporting latent correlations (Hardy et al., 2013, 2014; Yukhymenko-Lescroart and Sharma, 2020). In 23 instances (85%), the correlation coefficients were included in the published version of peer-reviewed journal articles. In four instances, authors of published peer-reviewed journal articles were contacted via email and provided the correlation coefficients or data to compute these (Cohen et al., 2011, 2014; Hardy et al., 2014; Hofmann et al., 2018). All correlation coefficients included in the present study were cross-sectional. Only a single study included a longitudinal analysis examining reciprocal links between moral identity and meaning in life over time to better understand temporal precedence of these variables (Han et al., 2019). This study found that moral identity predicts increased meaning in life over time, whereas meaning in life did not predict changes in moral identity suggesting that moral identity is a predictor of greater meaning in life but not vice versa.

### Overall effect size

**FIGURE 2:**
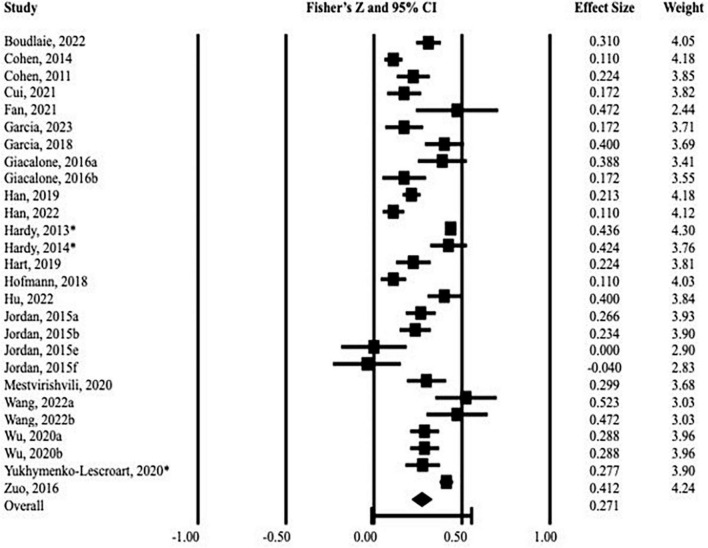
Forest plot of effect sizes with 95% confidence intervals for each study included in the main analysis. *Latent correlations.

For each study, the individual and pooled effect sizes of the association between moral identity and emotional well-being and its sub-dimensions are reported in Table 2. A Forest plot depicting the effect sizes of all included studies together with 95% confidence intervals is shown in Figure 2. The meta-analysis showed a significant overall correlation indicating that higher moral identity is associated with greater emotional well-being (*r* = 0.27, *p* < 0.001; CI_95%_ [0.22–0.33]). This indicates a medium effect size according to conventional effect size interpretation guidelines (Rice and Harris, 2005). There was a large proportion of heterogeneity (*I*^2^ = 93.42) and the variance of true effect sizes (τ^2^) was 0.02. Relatedly, the *Q*-test for heterogeneity indicated that effect sizes were heterogenous across studies supporting the use of a random effects model (*Q*_26_ = 395.15, *p* < 0.001). The *I*^2^ statistic had a score of 93, which indicates that 93% of the variance in observed effects reflected variance in true effects rather than sampling error. According to conventions, this *I*^2^ value is considered high (Higgins et al., 2003), meaning that the identified association is substantially due to true effects.

Results from the sensitivity analysis excluding three studies that reported latent correlations replicated the main results. The meta-analysis on the remaining 24 studies showed a significant overall correlation of medium size linking higher moral identity with greater emotional well-being (*r* = 0.25, *p* < 0.001; CI_95%_ [0.20–0.31]). As in the main analysis, effect sizes were heterogenous across studies (*Q*_23_ = 208.52, *p* < 0.001). However, heterogeneity decreased upon removal of latent effect sizes (*I*^2^ = 88.97, τ^2^ = 0.01). Results from a post-hoc sensitivity analyses showed that moral identity is related to greater emotional well-being even if the symbolization subscale of the SIMI is used with the effect size being similar (*r* = 0.22, *p* < 0.001) to when only the internalization subscale is used (*r* = 0.24, *p* < 0.001).

### Effect sizes by domain of emotional well-being

Results from the meta-analyses examining links between moral identity and each dimension of emotional well-being (i.e., happiness/positive affect, sense of purpose/meaning, life satisfaction, and self-esteem) are reported in Table 3. The results showed that moral identity is associated with more happiness and positive affect, a greater sense of purpose and meaning in life, more life satisfaction, and higher self-esteem. The effect sizes were medium for happiness/positive affect (*r* = 0.28, *p* < 0.001), sense of purpose/meaning in life (*r* = 0.29, *p* < 0.001), and self-esteem (*r* = 0.25, *p* < 0.001), and small for life satisfaction (*r* = 0.15, *p* = 0.011). Effect sizes were heterogenous between studies for each domain and between 84% and 95% of variance in observed effects reflected variance in true effects rather than sampling error. Since the sense of purpose/meaning and self-esteem domains both included two studies that reported latent correlations as the effect size, a sensitivity analysis was conducted excluding the latent effect sizes. The results from this sensitivity analysis replicated the original results by showing a medium effect size for moral identity and meaning/purpose (*r* = 0.26, *p* < 0.001) as well as a small to medium effect size for moral identity and self-esteem (*r* = 0.22, *p* < 0.001).

**TABLE 3:** Effect sizes of links between moral identity and individual dimensions of emotional well-being.

EWB dimension	No. of studies	Effect size (*r*)	*p*	CI 95% [LL, UL]	*Q* (df)	*I* ^2^
Happiness/positive affect	11	0.28	<0.001	[0.21, 0.36]	55.95 (10)	82.13
Sense of purpose/meaning	8*	0.29	<0.001	[0.21, 0.39]	117.9 (7)	94.07
Life satisfaction	6	0.15	0.011	[0.04, 0.27]	60.30 (5)	91.71
Self-esteem	9*	0.25	<0.001	[0.16, 0.36]	187.62 (8)	95.74

*Including two studies that report latent correlations.

### Cultural origin as a moderator

Results from a subgroup analysis showed a slightly larger effect size of moral identity and emotional well-being in studies conducted in more collectivistic countries (*r* = 0.30, *p* < 0.001) compared to studies conducted in more individualistic countries (*r* = 0.27, *p* < 0.001). However, the difference between these two effect sizes was not statistically significant (*Q*_1_ = 0.38, *p* = 0.536). It is important to note that all studies that report latent correlations were in the individualistic group, which may have attenuated differences between collectivistic and individualistic cultures. Therefore, a sensitivity analysis was conducted excluding these latent effect sizes. The results showed a larger difference in effect sizes with moral identity being more strongly related to emotional well-being in collectivistic (*r* = 0.30, *p* < 0.001) as compared to individualistic cultures (*r* = 0.23, *p* < 0.001). However, this difference was also not statistically significant (*Q*_1_ = 2.15, *p* = 0.142). A second sensitivity analysis was conducted that excluded two studies that were conducted in Iran and Georgia to enable a comparison between studies conducted in North-America (United States and Canada) and studies conducted in East and Southeast Asia (China and Indonesia). The results from this sensitivity analysis replicated the original results by showing slightly larger effect sizes in collectivistic Asian cultures (*r* = 0.30, *p* < 0.001) compared to individualistic Western cultures (*r* = 0.27, *p* < 0.001) but this difference was not statistically significant (*Q*_1_ = 0.32, *p* = 0.572). Similarly, when excluding latent correlations as well as the studies from Iran and Georgia, effect sizes are larger in the collectivistic group (*r* = 0.30, *p* < 0.001) versus in the individualistic group (*r* = 0.23, *p* < 0.001). However, this difference was not statistically significant (*Q*_1_ = 2.94, *p* = 0.087).

### Risk of bias

**FIGURE 3:**
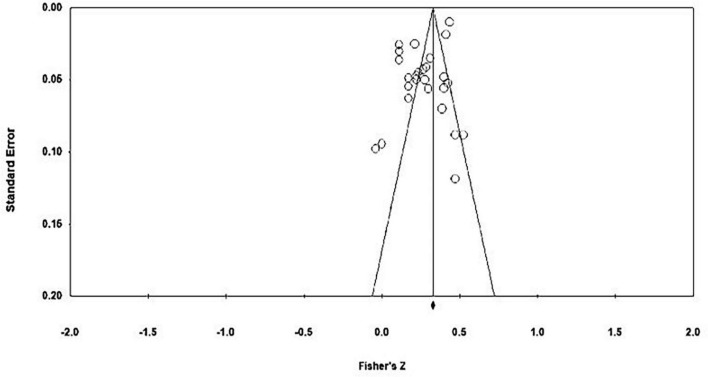
Funnel plot of standard error by Fisher’s *Z* of moral identity and overall emotional well-being including all studies, adapted from CMA 3.0 (Borenstein et al., 2022).

**FIGURE 4:**
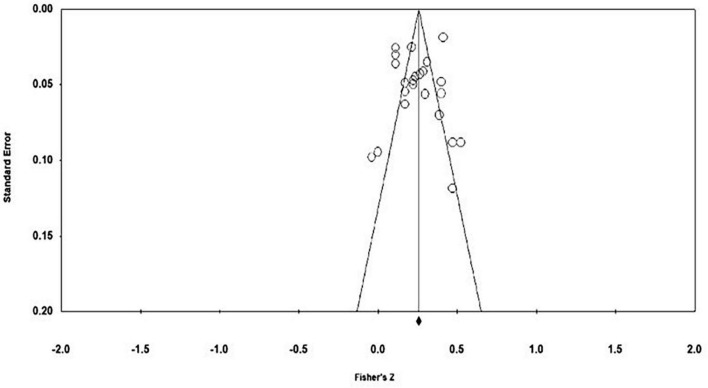
Funnel plot of standard error by Fisher’s *Z* of moral identity and overall emotional well-being excluding studies with latent correlations, adapted from CMA 3.0 (Borenstein et al., 2022).

Based on a funnel plot (see Figure 3), the distribution of effect sizes of links between moral identity and overall emotional well-being was not completely symmetrical around the average overall effect size. Egger’s regression intercept further indicated funnel plot asymmetry (*t* = 2.77, *p* = 0.010). However, the rank correlation test was not significant suggesting no asymmetry (τ = 0.09, *p* = 0.246). In the presence of publication bias, smaller studies would be expected to have larger effect sizes due to requiring larger effect sizes to reach statistical significance and smaller studies being more likely to get published if they produce significant results (Sterne and Harbord, 2004). However, the funnel plot does not provide any indication of a tendency that smaller studies would have larger effect sizes as the effect sizes of studies with larger standard errors (at the bottom of the plot) are scattered both below and above the average overall effect size. It is important to note that asymmetry within the funnel plot can be due to other factors beyond publication bias such as between-study heterogeneity and methodological differences between studies (Page et al., 2021b). Additionally, upon removal of the latent effect sizes, the funnel plot appeared symmetrical (see Figure 4) and Egger’s regression test was not significant indicating that there was no asymmetry (*t* = 0.28, *p* = 0.390). Likewise, the rank correlation test was not significant suggesting no asymmetry (τ = 0.11, *p* = 0.228). The results of the fail-safe *N* calculation showed that an additional 9,478 studies reporting no correlation of moral identity and emotional well-being (*r* < 0.01) would be needed for the estimated effect size to become non-significant (*p* < 0.05). Finally, publication bias may also have been reduced by including studies that did not examine links between moral identity and emotional well-being as a primary objective. In fact, more than half of the included studies did not formulate a hypothesis on links between moral identity and emotional well-being (*n* = 14). Thus, the included effect sizes from these studies should not have affected the study’s chance of publication, which has likely been more affected by whether the primary study aims were supported with significant results.

## Discussion

Since antiquity, philosophers have connected morality and virtuousness with individual well-being (McBeath and Webb, 2002; Kim, 2020). While several empirical studies have examined links between moral identity and various indicators of emotional well-being, no prior study has systematically reviewed and synthesized this literature. Thus, the objective of this meta-analysis was to survey all previous empirical studies on the relationship between moral identity and emotional well-being. Moreover, this study examined links between moral identity and individual dimensions of emotional well-being, specifically, happiness or positive affect, meaning or purpose in life, life satisfaction, and self-esteem. Ultimately, this study investigated if links between moral identity and overall emotional well-being differ across individualistic and collectivistic cultures. The results support a robust association between higher moral identity and greater emotional well-being that is present across sub-dimensions of emotional well-being. The present results also suggest that links between moral identity and emotional well-being exist in both individualistic and collectivistic cultures and are similar in magnitude.

The literature search yielded 27 eligible studies that examined links between moral identity and emotional well-being. A meta-analysis on these 27 effect sizes showed a medium and significant correlation between moral identity and emotional well-being of *r* = 0.27. This effect size is about one third of a standard deviation larger than the mean effect size of meta-analyses in the social sciences (Richard et al., 2003). Thus, these results provide robust evidence that considering moral values as an important part of one’s understanding of self is related to better emotional well-being. The effect size found in the present study is similar to the medium effect sizes found in previous meta-analyses examining moral identity in relation to moral behavior (*r* = 0.22) (Hertz and Krettenauer, 2016) and moral emotions (*r* = 0.32) (Lefebvre and Krettenauer, 2019). Interestingly, the medium effect size of moral identity in relation to emotional well-being found in the present study is larger than the small effect sizes reported in previous meta-analyses examining emotional well-being in relation to prosocial tendencies (*r* = 0.13) (Hui et al., 2020) and racial/ethnic identity (*r* = 0.17) (Smith and Silva, 2011). Comparing these effect sizes across studies, the present findings suggest that moral identity may be even more proximal to emotional well-being than overall prosociality and cultural demographic aspects of identity. If moral identity is more strongly related to emotional well-being than overall prosociality, this may support that moral identity does not only relate to emotional well-being through the benefits of more frequently engaging in prosocial behavior (Patrick et al., 2018). Instead, links between moral identity and emotional well-being may involve additional mechanisms such as higher self-consistency (Van Gils and Horton, 2019) and a greater sense of agency in identity development (Hardy et al., 2013).

The present findings support that links between moral identity and emotional well-being replicate across individual dimensions of emotional well-being, specifically, happiness or positive affect, sense of purpose or meaning in life, life satisfaction, and self-esteem. However, the present results show some variation in the magnitude of these associations across these individual dimensions of emotional well-being. Specifically, the results suggest medium effect sizes between moral identity and happiness or positive affect (*r* = 0.28), purpose or meaning in life (*r* = 0.29), and self-esteem (*r* = 0.25), whereas the association between moral identity and life satisfaction only evidenced a small effect size (*r* = 0.15). One explanation for this smaller effect size could be that life satisfaction involves a reflective assessment of life overall and being satisfied with life may imply a sense of stagnation in some individuals. This sense of stagnation may be less compatible with high moral identity providing consistent direction and ideals to pursue (Higgins-D’Alessandro and Power, 2005) as well as making individuals more resistant to injustices in their environment (O’Reilly et al., 2016). Thus, life satisfaction may be less pronounced in individuals with high moral identity as compared to self-esteem, purpose or meaning in life, and happiness or positive affect.

The present findings suggest that links between moral identity and greater emotional well-being are of similar magnitude in individualistic and collectivistic cultures. Previous research has raised concerns that the most-widely used measures of moral identity may be prone to cultural bias (Jia and Krettenauer, 2017). The present findings strengthen the conceptual validity of moral identity across cultural contexts and may suggest that existing measures of moral identity are not culturally biased when examining moral identity in relation to emotional well-being. In line with the hypothesis, the effect size was larger in studies involving samples from collectivistic countries, which would suggest that moral identity is more strongly related to emotional well-being in collectivistic as compared to individualistic cultures. Previous findings have shown that individuals from collectivistic cultures experience more positive emotions in response to outcomes that benefit others as compared to individuals from individualistic cultures (Stipek, 1998). As high moral identity involves a merge of self-interests and other oriented moral motives (Frimer and Walker, 2009), these findings may explain stronger links between moral identity and emotional well-being in collectivistic cultures. However, the magnitude of this difference between effect sizes was small and not statistically significant. A recent cross-cultural study involving 49 countries examined how valuing interdependent happiness and individual life satisfaction differs by country (Krys et al., 2023). While people in Asian countries including China and Iran value interdependent happiness most strongly over individual life satisfaction, people in the United States and Canada, as stereotypically individualistic countries, also value interdependent happiness higher than individual life satisfaction and more so than most countries in Western-Europe and South and Central America (Krys et al., 2023). Thus, if cultural differences in links between moral identity and emotional well-being are due to greater emphasis on interdependent emotional well-being in collectivistic cultures, the marginal difference observed in the present results may be explained by the relatively small difference in how interdependent happiness is valued between the individualistic and collectivistic countries included in this review. Nevertheless, these results may provide a starting point for future cross-cultural research to explicitly investigate cultural differences in the role of moral identity in emotional well-being.

The present review showed that one third of the studies examining moral identity in relation to emotional well-being were conducted in countries that are considered collectivistic (Minkov and Kaasa, 2022). Thus, the proportion of included studies from more collectivistic countries in the present review is substantially larger than in previous meta-analytic reviews (Hertz and Krettenauer, 2016; Lefebvre and Krettenauer, 2019). However, the majority of eligible studies were conducted in the United States and China, which resulted in little cultural diversity within the individualistic and collectivistic groups. The eligible studies also showed little diversity in how moral identity was measured as all but one study assessed moral identity with the Self-Importance of Moral Identity Scale (Aquino and Reed, 2002). Similarly, the eligible studies showed little diversity in how self-esteem and life satisfaction were measured as all eligible studies measured self-esteem with Rosenberg’s Self-Esteem Scale (Rosenberg, 1965) and life satisfaction with the original Satisfaction with Life scale (Diener et al., 1985) or a related version (Pavot et al., 1998). Future studies may therefore examine alternative measures of moral identity in relation to emotional well-being and utilize different measures of self-esteem and life satisfaction. While the included studies were generally diverse in terms of age and gender, no previous study examined links between moral identity and emotional well-being in older adult populations. Since moral concerns are particularly salient in older adults (Narvaez et al., 2011) it could be an interesting avenue for future research to investigate the role of moral identity in the emotional well-being of older adults. Finally, the vast majority of previous studies examined links between moral identity and emotional well-being only cross-sectionally, which did not enable a testing of temporal precedence. Only one study provided evidence of moral identity predicting changes in meaning of life over time (Han et al., 2019). No longitudinal studies have examined moral identity as a predictor of life satisfaction, self-esteem, or happiness and positive affect. Thus, further longitudinal studies are needed that incorporate various dimensions of emotional well-being to better understand if moral identity predicts greater emotional well-being and can be a target for intervention efforts designed to improve emotional well-being.

### Limitations and implications

Several limitations need to be considered when interpreting the results of the present study. Most importantly, the meta-analysis was based on correlational effect sizes and therefore did not examine temporal precedence. Thus, any directional inferences cannot be made based on the present results. A second limitation is that only a few studies involved older adult samples and the results may therefore not be generalizable to older adult populations. Even though this review included studies from diverse cultural backgrounds, the vast majority of studies were conducted in the United States and China, which may suggest that results are less generalizable to individuals from other parts of the world. Relatedly, the analysis examining the moderating role of culture is limited by focusing on individualism and collectivism, which by no means captures every aspect of cultural difference between the studies in the individualistic and collectivistic groups (Brewer and Chen, 2007). Similarly, the present study could not consider cultural differences between the countries within the individualistic and collectivistic groups or at the participant level. Another limitation may be that the sample size did not allow for separate analyses of culture as a moderator in each individual relationship of moral identity and the four dimensions of emotional well-being.

Since this study involved study-level data with many studies showing little variability in gender composition, gender differences in links of moral identity and emotional well-being could not be examined. A previous meta-analysis showed that females have higher moral identity compared to males (Kennedy et al., 2017) but none of the empirical studies included in this review conducted a formal statistical analysis on gender differences in links between moral identity and emotional well-being. However, one study examined links between moral identity and life satisfaction separately by gender reporting the same effect size (*r* = 0.28) for males and females (Wu et al., 2020). Nevertheless, a more thorough investigation on whether links between moral identity and emotional well-being differ by gender is an interesting direction for future research.

Although this meta-analysis was based on correlational results and existing longitudinal evidence is limited it is possible that promoting moral identity may be a pathway to achieve greater emotional well-being. Thus, the present findings highlight the need of longitudinal studies to better understand the directionality of this relationship and examine if existing programs designed to promote moral identity also benefit individuals’ emotional well-being. Examples of successful interventions include promoting moral identity through stories about behaviors of moral exemplars (Čehajić-Clancy and Olsson, 2023). Additionally, moral identity may be fostered through community engagement and service learning (Narvaez and Lapsley, 2009) which have also been found to increase emotional well-being (Lawton et al., 2021). If moral identity indeed predicts better emotional well-being, promoting moral identity through these programs may become a relevant target for interventions designed to improve various dimensions of emotional well-being (Kubzansky et al., 2023).

## Conclusion

The current review and meta-analysis examined previous studies on the relationship between moral identity and emotional well-being together with differences across domains of emotional well-being and between collectivistic and individualistic cultures. In summary, the present results support the idea that individuals who consider moral values as central to their self-identity are experiencing greater emotional well-being in form of happiness and positive affect, meaning and purpose in life, higher self-esteem, and life satisfaction to a smaller degree. Additionally, the present findings show that moral identity is similarly related to emotional well-being in collectivistic and individualistic cultures. Thus, moral identity may not only benefit moral functioning but also a person’s emotional well-being across cultures. However, it is important to note that the present findings are correlational and therefore do not allude to any temporal precedence or causal inference. Thus, further longitudinal and experimental studies are needed to understand if fostering moral identity could be a pathway to increase emotional well-being and achieve what Aristotle defined as eudaimonia.

## Data availability statement

The datasets presented in this study can be found in online repositories. The names of the repository/repositories and accession number(s) can be found below: https://osf.io/94f8b/?view_only=6db54da0fa304c83993d0438ecb5c637.

## Author contributions

MG: Conceptualization, Data curation, Formal analysis, Investigation, Methodology, Visualization, Writing – original draft, Writing – review & editing. CE: Data curation, Formal analysis, Investigation, Methodology, Visualization, Writing – original draft, Writing – review & editing. AM: Formal analysis, Investigation, Methodology, Software, Visualization, Writing – original draft. EE: Data curation, Investigation, Visualization, Writing – original draft. CJ: Data curation, Investigation, Visualization, Writing – original draft. CR: Formal analysis, Methodology, Software, Supervision, Funding acquisition, Project administration, Writing – review & editing.

## Footnotes

1
https://osf.io/94f8b/?view_only=6db54da0fa304c83993d0438ecb5c637

2
https://www.hofstede-insights.com/country-comparison-2023
